# Variation in the Soil Prokaryotic Community Under Simulated Warming and Rainfall Reduction in Different Water Table Peatlands of the Zoige Plateau

**DOI:** 10.3389/fmicb.2020.00343

**Published:** 2020-03-18

**Authors:** Wei Li, Huai Chen, Zhiying Yan, Gang Yang, Junpeng Rui, Ning Wu, Yixin He

**Affiliations:** ^1^Key Laboratory of Mountain Ecological Restoration and Bioresource Utilization and Ecological Restoration Biodiversity Conservation Key Laboratory of Sichuan Province, Chengdu Institute of Biology, Chinese Academy of Sciences, Chengdu, China; ^2^Zoige Peatland and Global Change Research Station, Chinese Academy of Sciences, Hongyuan, China; ^3^School of Ecology and Environmental Sciences and Yunnan Key Laboratory for Plateau Mountain Ecology and Restoration of Degraded Environments, Yunnan University, Kunming, China; ^4^Center for Excellence in Tibetan Plateau Earth Sciences, Chinese Academy of Sciences, Beijing, China; ^5^School of Life Sciences and Engineering, Southwest University of Science and Technology, Mianyang, China

**Keywords:** prokaryotic communities, climate change, water table, dry-rewetting event, 16S rRNA gene sequencing

## Abstract

Climate change and water table drawdown impact the community structure and diversity of peatland soil prokaryotes. Nonetheless, how soil prokaryotes of different water tables respond to climate change remains largely unknown. This study used 16S rRNA gene sequencing to evaluate the variation in soil prokaryotes under scenarios of warming, rainfall reduction, and their combination in different water table peatlands on the Zoige Plateau in China. Stimulated climate change affected some of the diversity indexes and relative abundances of soil prokaryotes in three water table peatlands. Additionally, those from the dry-rewetting event peatland had the most dominant phyla (genera) that showed significant changes in a relative abundance due to the simulated climate change treatments. Regarding functional microbial groups of carbon and nitrogen cycling, simulated climate change did not affect the abundances of the Euryarchaeota, Proteobacteria, Verrucomicrobia, and *Methanobacterium* in three water table peatlands, except NC10 and Nitrospirae. Redundancy analysis showed that the prokaryotic community variation was primary impacted by site properties of the different water table peatlands rather than the simulated climate change treatments. Moreover, the water table, total carbon, total nitrogen, and soil pH were the primary factors for the overall variation in the soil prokaryotic structure. This study provides a theoretical guidance for management strategies in the Zoige peatland, under climate change scenarios. More attention should be given to the interactive effects of peatland water table drawdown and simulated climate changes for better restorative efforts in water table drawdown, rather than simply adapting to climate change.

## Introduction

Warming and fluctuations in rainfall patterns are common aspects of global climate change around the world, especially in the Qinghai-Tibetan Plateau (Liu and Chen, 2000; Yao et al., 2000), which is becoming warmer (Liang et al., 2009; Chen et al., 2013) and slightly drier (Yang et al., 2014). In the eastern Qinghai-Tibetan Plateau, the Zoige peatland faces water table drawdown and degradation due to climate change, overgrazing, and land reclamation for livestock (Wang et al., 2011; Guo et al., 2013). The combined effects of peatland degradation and climate change are likely to have a significant impact on soil conditions (Shang et al., 2013). The soil microbial communities are an essential component of soil ecosystems, as they are responsible for carbon and nitrogen cycling, and ecosystem processes (Falkowski et al., 2008). The microbial community structure is directly related to environmental factors (Rinnan et al., 2007; Deslippe et al., 2012; Corneo et al., 2014) and soil conditions (Brockett et al., 2012; Dennis et al., 2013; Zhang X.M. et al., 2014). Therefore, it is important to understand how climate change and peatland water table drawdown affects the structure and biogeochemical cycling of soil microbial communities, which will help predicate future management strategies.

Many *in situ* experiments have studied the response of soil microbial communities to warming (Walker et al., 2008; Rinnan et al., 2011; Schindlbacher et al., 2011; Yergeau et al., 2011; Deslippe et al., 2012; Corneo et al., 2014; Xiong et al., 2014; Zhang B. et al., 2014), but fewer studies have analyzed the response of soil microbes to rainfall reduction (Cregger et al., 2012), or the combined effect of warming and rainfall reduction (Bérard et al., 2011; Zhang et al., 2013; Jumpponen and Jones, 2014). For the Qinghai-Tibetan Plateau, only a handful of studies have examined the potential impact of warming and rainfall changes on the community structure, abundance, and diversity of soil microbes (Zheng et al., 2012; Zhang et al., 2013; Zhang B. et al., 2014; Xiong et al., 2014). Zhang et al. (2013) reported that warming did not change the soil microbial community structure in semi-arid grasslands, which may be due to the fact that these microbial communities have already adapted to fluctuating climatic conditions (Zhang et al., 2013). However, other studies have found a strong influence of warming on soil microbial communities (Zheng et al., 2012; Xiong et al., 2014; Zhang B. et al., 2014). Peatland warming studies have shown that warming destabilized the carbon and nutrient recycling via changes in the microbial food web (Jassey et al., 2013), and that the microbiota responded to increasing temperature by modulating their metabolic and trophic interactions, which increased methane production (Tveit et al., 2015). To date, no consensus has emerged on how soil organisms will respond to warming and precipitation change. Worldwide peatland degradation is predicted to increase by 17% from 2008 to 2025 (Urák et al., 2017), with one vital phenomenon for peatland degradation being water table drawdown. Studies have shown that the overall composition of prokaryotic and eukaryotic communities, aerobic decomposers, and methane cycle microbes are affected by drought and rewetting (Potter et al., 2017), water table drawdown (Jaatinen et al., 2007; Yrjälä et al., 2011), and drainage (Jaatinen et al., 2005). However, there remains to be a systematic study that investigates the effect of simulated climate changes on soil microorganisms in different water table peatlands, especially on the Qinghai-Tibetan Plateau.

This research aimed to (1) verify whether simulated climate change affects prokaryotic communities and (2) explore whether the response of soil prokaryotes to simulated climate change was consistent among different water table peatlands.

## Materials and Methods

### Site Description

**FIGURE 1:**
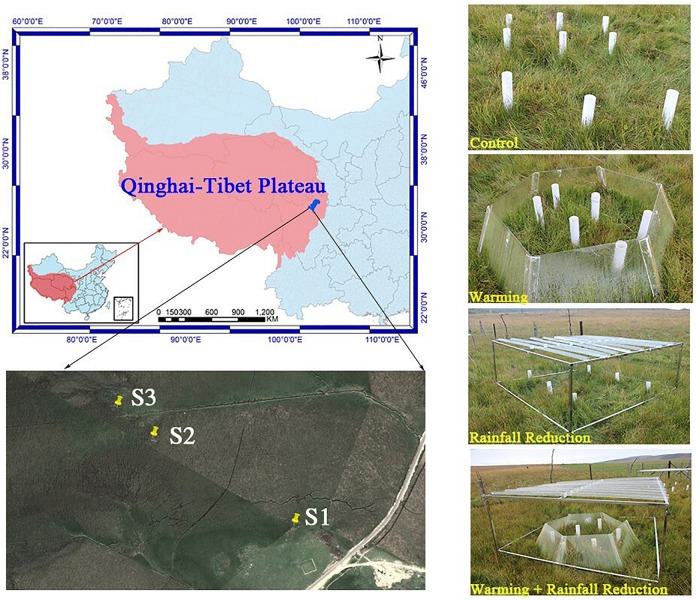
Map of study sites, showing the location and appearance of the simulated climate change treatments.

The experimental sites were located in the Riganqiao Peatland Nature Reserve (3471 m a.s.l., 33°06′15′′ N, 102°39′08′′ E) in Hongyuan County of the northwestern Sichuan Province, China (Figure 1). Hongyuan County has a cold temperate and continental plateau monsoon climate, with an annual average temperature of 1.1°C and an extreme minimum temperature of −36.2°C. The annual rainfall is 753 mm. The Riganqiao Peatland Nature Reserve, an important part of the Zoige peatland, is a high-altitude fen (Yang et al., 2019) with a total area of 1075 km^2^.

### Experimental Design

Three peatland sites (S1, S2, and S3) with characteristic plant community and various water tables were selected for further evaluation (Supplementary Figure S1). For S1, the lowest and highest monthly water table averages were −38.01 and −9.17 cm, respectively. *Equisetum ramosissimum* Desf was the dominant species, and the harvested aboveground biomass in 20 cm × 20 cm quadrats was 3.60 kg/m^2^. For S2, with an annual dry-rewetting cycle, their lowest monthly water table average was −19.76 cm and was waterlogged in July. *Caltha palustris* was the dominant species, and the harvested aboveground biomass was 2.41 kg/m^2^. S3 is saturated year-round; the lowest and highest monthly water table averages were −4.88 and 1.93 cm, respectively. *Carex mulienses* was the dominate species, and the aboveground biomass was 4.19 kg/m^2^.

We established a complete factorial design with four treatments in each of the three sites of peatlands in July 2012: control (CK: ambient temperature and ambient rainfall), warming (O: passive warming with open-top chambers), 20% rainfall reduction (R: reduced rainfall with a rainout shelter), and a combined warming and 20% rainfall reduction (RO: passive warming with open-top chambers plus 20% rainfall reduction with a rainout shelter). Three replicates were taken from each treatment plot, and thus the total samples were 36 plots.

The open-top chamber for passive warming was a transparent hexagon PVC chamber that consisted of six equal-sized trapezoids, with a dominant open area of 1.273 m^2^, bottom open area of 2.598 m^2^, and a vertical height of 0.52 m. The rainout shelter was composed of metal frames (1.3 m × 1.3 m) supporting 8 V-shaped clear acrylic bands (1.3 m × 0.125 m) to reduce the total rainfall by about 20% of the ambient level.

### Sample Collection and Preliminary Analysis

For each treatment plot, four soil samples were collected by cores with 8 cm in diameter and 15 cm in height in November 2013, and then were mixed to one composite sample. Thus, 36 composite samples were transported on ice to the Chengdu Institute of Biology and stored at −20°C until processing. Soil samples were air-dried, and sieved at 2 mm for pH analysis and 0.15 mm (mesh) for total carbon (TC) and total nitrogen (TN). Approximately 0.05 g of each sieved sample was used to measure TC and TN with a CN Analyzer (Multi N/C 2100s, Jena, Germany). Soil pH was measured in a soil/water suspension (1:5). Soil temperature at 5 cm depth was measured with a WatchDog B-Series Docking Station (Spectrum Technologies, Inc., Plainfield, IL, United States; data sampling frequency = 1 h) for all of November. Peat mass moisture contents were measured using the oven drying method.

### DNA Extraction, 16S rRNA Gene Amplification, and Sequencing

DNA was extracted from 0.5 g of composite soil using the Omega E.Z.N.A^TM^ Soil DNA Kit (Omega Bio-Tek, Inc., Norcross, GA, United States) according to the manufacturer’s instructions. The V4 region of the 16S rRNA gene was PCR amplified in triplicate using the following primers that targeted bacteria and archaea: 515F = (5′-GTGCCAGCMGCCGCGGTAA-3′) and 806R = (5′-GGACTACHVGGGTWTCTAAT-3′).

The primers were tailed with sequences to incorporate Illumina adapters with indexing barcodes. The PCR details and the related procedures were based on the 16S rRNA Amplification Protocol version 4-13 (Caporaso et al., 2012). The DNA concentrations were quantified, and the amplicons from each sample were pooled with an equimolar concentration for sequencing on an Illumina MiSeq Sequencer (San Diego, CA, United States) at the Chengdu Institute of Biology, Chinese Academy of Sciences, China.

### Sequencing Data Processing

The samples were classified based on the unique sample barcodes, and then the raw sequences were trimmed with the QIIME software (Caporaso et al., 2010). The barcodes, primer sequences, and chimeras were also removed (Edgar et al., 2011), and the unqualified sequences were discarded. Operational taxonomic units (OTUs) were classified using 97% of the 16S rDNA gene sequence similarity as a cutoff (Kuczynski et al., 2012). Each sample was rarefied to the same number of reads (10,280 sequences) for alpha-diversity (Chao 1 estimator of richness, observed species, and Shannon’s diversity index) analysis. The phylogenetic affiliation of each sequence was analyzed with the RDP Classifier at a confidence level of 80%^1^.

### Statistical Analysis

We carried out the redundancy analysis (RDA) to investigate the relationship between soil prokaryotic community and environmental variables (water table, soil temperature, TC, TN, and pH) under three water table peatlands and the simulated climate change treatments by running the RDA in Canoco (Version 4.5 for Windows, Petr and Jan, 2004). PerMANOVA was used to test the statistical differences between data sets in PAST using the weighted UniFrac distance^2^. The Mantel test was performed to evaluate the relationship between the prokaryotic community and the environmental variables using the R package Vegan. To determine the differences in prokaryotic communities between different water table peatlands, ANOVA was used to analyze the soil prokaryotic community diversity and dominant abundances among the control treatments. To evaluate how the soil prokaryotic microbes respond to simulated climate change, ANOVA was used to analyze the differences in the soil prokaryotic community diversity and dominant abundances between the control and treatment groups. Least significant difference (LSD) was performed for each variable. The ANOVA and LSD were applied based on the assumptions of a normal distribution and homogeneity of variance. Pearson’s correlation analysis was used to examine the correlation between each genus and environmental variables using the SPSS 16.0 software.

## Results

### Soil Temperature and Moisture

In November 2013, after more than 1 year of the simulated warming and rainfall reduction treatments, the monthly average temperature at 5 cm peat depth was −0.20 to 2.20°C in the S1 peatland, 0.07 to 1.85°C in the S2 peatland, and −0.61 to 0.81°C in the S3 peatland. In the S1, after treatment of warming, 20% rainfall reduction, or their combination, the soil temperature at 5-cm peat depth increased by an average of 0.96, 0.23, and 2.4°C, respectively. The soil temperature increase at 5-cm depth in the S2 was 1.61, 0.32, and 1.78°C, respectively, and for the S3, 1.43, 0.61, and 1.33°C, respectively (Supplementary Table S1). We measured peat moisture and found that the effect of the rainfall reduction treatment was dependent on the water table of each peatland. In the S1, the peat moisture in the control and rainfall reduction treatments were 42 and 29%, respectively, indicating that the rainfall reduction worked to significantly reduce the moisture. The S3 is saturated year-round, and the S2 keeps an annual dry-rewetting cycle in the non-growing and growing season, respectively. Thus, the rainfall reduction in the S2 and S3 did not affect the peat moisture because of the high water table (Supplementary Table S1).

### Variation in the Soil Prokaryotic Communities of Different Water Table Peatlands Under Simulated Climate Change

**FIGURE 2:**
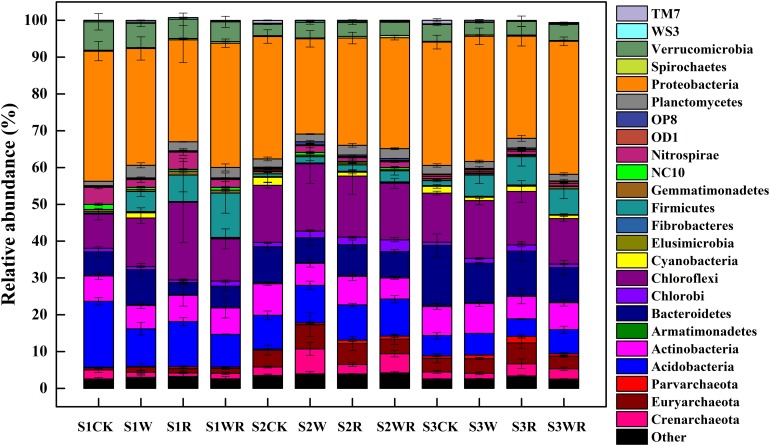
Differences in the relative abundances of the soil prokaryotic communities at the phylum level in peatlands of different water table under simulated climate change.

Our analysis showed that the most abundant phylum in the Zoige peatland was Proteobacteria (average relative abundance of 25.89–36.14%), followed by Acidobacteria, Actinobacteria, Bacteroidetes, Chloroflexi, and Verrucomicrobia, with average relative abundances from 3.10 to 21.13% (Figure 2). The dominant genera were *Candidatus Solibacter*, *Methanobacterium*, *Hyphomicrobium*, *Rhodoplanes*, and *DA101* (Supplementary Table S2).

In the S1, warming significantly increased the Chao1 value, the number of observed species, and Shannon’s diversity index, while the 20% rainfall reduction and the combined treatment of warming and rainfall reduction did not significantly influence any of the three indices (Table 1 and Supplementary Table S3). In the S2, warming significantly decreased the number of observed species and Shannon’s diversity index, but the combined warming and rainfall reduction treatment significantly decreased the number of observed species and increased Shannon’s diversity index (Table 1 and Supplementary Table S3). In the S3, warming, rainfall reduction, and their combination had no significant effect on the soil prokaryote diversity (Table 1 and Supplementary Table S3).

**TABLE 1:** ANOVA results for the response of Chao 1, observed species, and Shannon index to the different treatments (*P*-value as compared to the control).

Gradients Treatments	S1			S2			S3		
	W	R	WR	W	R	WR	W	R	WR
Chao1 estimator of richness	0.046*	0.359	0.483	0.111	0.836	0.223	0.768	0.856	0.410
Observed species	0.026*	0.411	0.516	0.022*	0.349	0.048*	0.503	0.903	0.570
Shannon’s diversity index	0.018*	0.300	0.393	0.009**	0.275	0.036*	0.639	0.594	0.813

W: warming; R: 20% rainfall reduction; WR: warming + 20% rainfall reduction. *P < 0.05; **P < 0.01.

Warming, 20% rainfall reduction, and their combination significantly affected the abundances of some communities (24 dominant phyla and 13 dominant known genera) in the three categories of water table peatlands (Figure 2, Table 2, and Supplementary Table S2). Thirteen dominant phyla (genera) were sensitive to the simulated climate change in the S1, 23 in the S2, and 7 in the S3. In terms of the functional microbes for carbon and nitrogen cycling, the abundances of the Euryarchaeota, Proteobacteria, and Verrucomicrobia phyla in three water table peatlands were not affected by the stimulated climate change treatments, while NC10 and Nitrospirae had no uniform or consistent response (Table 2). The abundance of the *Methanobacterium* genus also had no significant change (Table 2). There was a significant difference of observed species and Shannon’s diversity among three water table peatlands on the control, stimulated warming, and rainfall reduction treatments. For Chao1, there was a significant difference among three water table peatlands on the stimulated warming, rainfall reduction, and their combined effect (Table 3). Of the 24 dominant phyla and 13 genera, 18 phyla (genera) showed significant differences in their relative abundance in the control treatment, 8 were significantly different after the warming treatment, 6 differed after the 20% rainfall reduction, and 9 differed after the combined treatment (Table 4). For the functional microbes for carbon and nitrogen cycling, the relative abundances of Euryarchaeota, Proteobacteria, and Verrucomicrobia did not change in three water table peatlands, but the abundances of NC10 and Nitrospirae changed significantly (Table 4). The relative abundances of Verrucomicrobia, NC10, and Nitrospirae were highest in the S1 (Figure 2). The relative abundance of *Methanobacterium* significantly varied among the three water table peatlands and was highest in the S2 (Supplementary Table S2).

**TABLE 2:** ANOVA results for the responses of the abundance of dominant bacteria and archaea at phylum and genus levels to the different treatments in the different water table peatlands (*P*-values as compared to the control).

	S1			S2			S3		
Treatment	W	R	WR	W	R	WR	W	R	WR
**(a) Phylum**									
Crenarchaeota	0.252	0.114	0.288	0.066	0.651	0.031*	0.503	0.356	0.253
Euryarchaeota	0.401	0.454	0.136	0.603	0.673	0.749	0.836	0.366	0.825
Parvarchaeota	0.517	0.258	0.199	0.418	0.074	0.122	0.193	0.074	0.917
Acidobacteria	0.018*	0.019*	0.001**	0.574	0.782	0.609	0.720	0.458	0.480
Actinobacteria	0.677	0.935	0.922	0.076	0.543	0.007**	0.855	0.466	0.683
Armatimonadetes	0.550	0.879	0.067	0.099	0.437	0.058	0.082	0.126	0.412
Bacteroidetes	0.218	0.089	0.765	0.284	0.438	0.061	0.156	0.367	0.131
Chlorobi	0.652	0.379	0.241	0.013*	0.037*	0.022*	0.101	0.163	0.536
Chloroflexi	0.148	0.350	0.535	0.639	0.836	0.932	0.138	0.724	0.377
Cyanobacteria	0.353	0.344	0.136	0.174	0.460	0.185	0.193	0.490	0.215
Elusimicrobia	0.427	0.067	0.329	0.814	0.185	0.649	0.462	0.181	0.899
Fibrobacteres	0.221	0.259	0.930	0.692	0.606	0.237	0.507	0.275	0.431
Firmicutes	0.022*	0.210	0.099	0.408	0.499	0.124	0.126	0.069	0.103
Gemmatimonadetes	0.339	0.435	0.562	0.321	0.670	0.088	0.143	0.220	0.160
NC10	0.123	0.098	0.203	0.169	0.087	0.061	0.009**	0.071	0.024*
Nitrospirae	0.037*	0.998	0.022*	0.075	0.033*	0.007**	0.100	0.191	0.755
OD1	0.912	0.260	0.449	0.866	0.870	0.798	0.813	0.783	0.804
OP8	0.430	0.769	0.181	0.219	0.425	0.084	0.046*	0.226	0.492
Planctomycetes	0.031*	0.000**	0.158	0.766	0.592	0.470	0.464	0.791	0.417
Proteobacteria	0.428	0.317	0.604	0.064	0.163	0.181	0.873	0.152	0.301
Spirochaetes	0.927	0.656	0.200	0.936	0.065	0.020*	0.226	0.363	0.766
Verrucomicrobia	0.664	0.424	0.430	0.093	0.258	0.012*	0.034*	0.588	0.612
WS3	0.970	0.612	0.183	0.482	0.446	0.304	0.730	0.463	0.154
TM7	0.025*	0.524	0.006**	0.011*	0.072	0.008**	0.296	0.102	0.174
**(b) Genera**									
*Methanobacterium*	0.102	0.098	0.098	0.136	0.757	0.226	0.236	0.415	0.462
*Candidatus Solibacter*	0.498	0.198	0.113	0.033*	0.046*	0.031*	0.438	0.286	0.388
*Arthrobacter*	0.344	0.629	0.196	0.625	0.152	0.010*	0.589	0.161	0.012*
*Hyphomicrobium*	0.322	0.580	0.354	0.371	0.737	0.021*	0.395	0.476	0.067
*Rhodoplanes*	0.650	0.677	0.144	0.622	0.981	0.513	0.174	0.300	0.032*
*Anaeromyxobacter*	0.142	0.067	0.025*	0.006**	0.512	0.001**	0.582	0.741	0.578
*Desulfobacca*	0.058	0.123	0.627	0.124	0.007**	0.277	0.292	0.023*	0.076
*Desulfomonile*	0.353	0.882	0.734	0.520	0.786	0.084	0.114	0.539	0.919
*Cellvibrio*	0.434	0.137	0.036*	1.000	1.000	0.374	0.394	0.519	0.148
*Pseudomonas*	0.285	0.632	0.949	0.839	0.363	0.186	0.228	0.312	0.780
*Steroidobacter*	0.117	0.022*	0.070	0.000**	0.002*	0.001**	0.101	0.868	1.000
*Luteolibacter*	0.684	0.727	0.064	0.603	0.623	0.001**	0.230	0.193	0.231
*DA101*	0.127	0.367	0.235	0.219	0.604	0.210	0.470	0.720	0.297

W: warming; R: 20% rainfall reduction; WR: warming + 20% rainfall reduction. *P < 0.05; **P < 0.01.

**TABLE 3:** ANOVA results for the response of Chao 1, observed species, and Shannon index to the simulated climate change treatments among the three levels of water table peatlands.

Treatment	Chao1 estimator of richness	Observed species	Shannon’s diversity index
CK	0.122	0.025*	0.021*
W	0.050*	0.013*	0.004**
R	0.007*	0.018*	0.046*
WR	0.038**	0.053	0.137

CK: no warming or 20% rainfall reduction; W: warming; R: 20% rainfall reduction; WR: warming + 20% rainfall reduction. *P < 0.05; **P < 0.01.

**TABLE 4:** ANOVA results for the responses of the dominant bacteria and archaea at the phylum and genus levels to the simulated climate change treatments among the three levels of water table peatlands.

Treatment	CK	W	R	WR
**(a) Phylum**				
Crenarchaeota	0.757	0.019*	0.183	0.012*
Euryarchaeota	0.080	0.259	0.054	0.105
Parvarchaeota	0.067	0.021*	0.094	0.486
Acidobacteria	0.000**	0.066	0.001**	0.051
Actinobacteria	0.353	0.257	0.871	0.773
Armatimonadetes	0.043*	0.791	0.861	0.359
Bacteroidetes	0.037*	0.282	0.031*	0.289
Chlorobi	0.016*	0.026*	0.088	0.013*
Chloroflexi	0.016*	0.581	0.809	0.351
Cyanobacteria	0.200	0.502	0.027*	0.086
Elusimicrobia	0.775	0.768	0.061	0.710
Fibrobacteres	0.957	0.212	0.223	0.205
Firmicutes	0.025*	0.229	0.384	0.274
Gemmatimonadetes	0.556	0.605	0.300	0.135
NC10	0.007**	0.198	0.311	0.080
Nitrospirae	0.000**	0.159	0.004**	0.080
OD1	0.130	0.152	0.070	0.601
OP8	0.193	0.193	0.872	0.002**
Planctomycetes	0.142	0.107	0.956	0.484
Proteobacteria	0.759	0.151	0.959	0.015*
Spirochaetes	0.662	0.502	0.633	0.103
Verrucomicrobia	0.109	0.026/	0.666	0.423
WS3	0.171	0.359	0.249	0.059
TM7	0.098	0.365	0.315	0.953
**(b) Genera**				
*Methanobacterium*	0.048*	0.228	0.122	0.441
*Candidatus Solibacter*	0.041*	0.734	0.345	0.630
*Arthrobacter*	0.000**	0.444	0.210	0.031*
*Hyphomicrobium*	0.029*	0.042*	0.134	0.000**
*Rhodoplanes*	0.015*	0.350	0.223	0.044*
*Anaeromyxobacter*	0.108	0.017*	0.268	0.002**
*Desulfobacca*	0.006**	0.032*	0.002**	0.130
*Desulfomonile*	0.012*	0.911	0.391	0.063
*Cellvibrio*	0.000**	0.096	0.306	0.115
*Pseudomonas*	0.137	0.007**	0.814	0.858
*Steroidobacter*	0.036*	0.003**	0.213	0.442
*Luteolibacter*	0.651	0.512	0.711	0.344
*DA101*	0.030*	0.073	0.048*	0.034*

CK: no warming or 20% rainfall reduction; W: warming; R: 20% rainfall reduction; WR: warming + 20% rainfall reduction. *P < 0.05; **P < 0.01.

**FIGURE 3:**
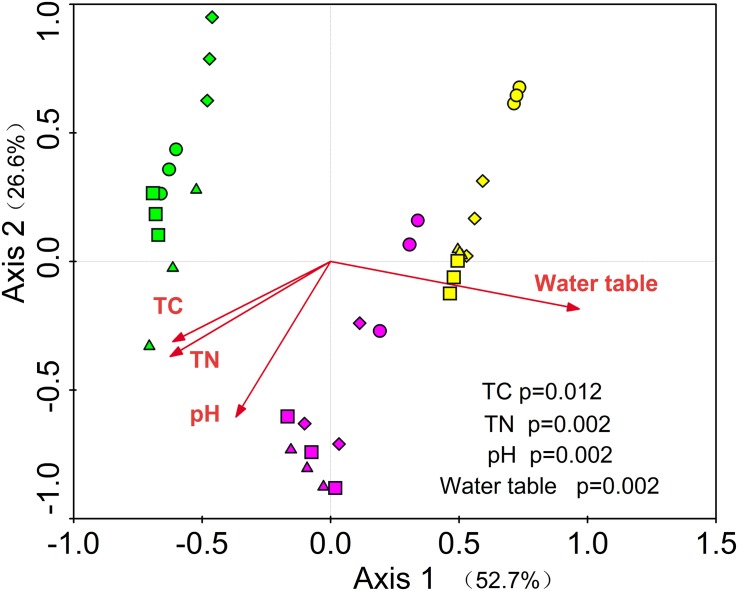
RDA of the relationships among soil prokaryotic community and environmental variables. Green = S1, purple = S2 and yellow = S3, circle = CK; diamond = R; up triangle = W; down triangle = WR.

The prokaryotic community variation was primarily impacted by different water table peatlands rather than the simulated climate change treatments (Figure 3 and Supplementary Table S4). The PerMANOVA test based on the UniFrac distance measures showed that the overall prokaryotic community structure was significantly different among the clusters grouped by different water table peatlands (*P* < 0.01, Supplementary Table S4). This was further supported by the fact that the change in the prokaryotic community structure was significantly correlated with total carbon, total nitrogen, pH, and the water table (*P* < 0.01, Table 5).

**TABLE 5:** Pearson’s correlation of the environmental variables and the prokaryotic community structure, as determined by the Mantel Test^a^.

Variable	Pearson correlation coefficient (*r*)	*P*
Total carbon	0.3757	< 0.01
Total nitrogen	0.3866	< 0.01
pH	0.1980	<0.01
Water table level	0.5604	< 0.01
Soil temperature	0.1296	< 0.05

*^a^Permutations, 9999 (using the relative abundances of OTU as input in analysis).*

### Correlations Between Soil Prokaryotic Communities and Environmental Variables

In general, the total carbon and nitrogen content did not change after the climate change treatments in the S2 and S3, while in the S1, warming, rainfall reduction, and their combination all significantly increased the soil carbon and nitrogen content (Supplementary Table S1). Soil pH increased significantly with warming, rainfall reduction, and their combination in all three peatlands (Supplementary Table S1). Soil total carbon, nitrogen, and pH showed the positive correlations with temperature (Supplementary Table S5). The total carbon and nitrogen content varied significantly among the peatlands of three water tables (S2 > S1 > S3). S2 had the highest pH value, and it was significantly different from that in S3 (Supplementary Table S1).

Redundancy analysis showed that the first and second axis explained the 52.7 and 26.6% variance of soil prokaryote community, respectively. Among these variables, total carbon (*P* = 0.012), total nitrogen (*P* = 0.002), water table (*P* = 0.002), and pH (*P* = 0.002) were the important determining factors (Figure 3). Among the dominant phyla (genera), we found 40 significantly correlated pairs of a phylum or genus with the soil properties or water table (*P* < 0.05), but only 12 correlated pairs with the soil temperature (*P* < 0.05, Supplementary Table S6).

## Discussion

### The Effect of Simulated Climate Change on the Soil Prokaryotic Communities

Our experiment found that small-scale (0.23–2.4°C) and short-term (1 year) warming changed the abundances of some of the groups (phylum and genus level) in the S1, S2, and S3 (Table 2). This is consistent with many previous short-term (<3 years) (Yergeau et al., 2011; Xiong et al., 2014; Zhang B. et al., 2014) and long-term studies (>10 years) (Rinnan et al., 2007; Deslippe et al., 2012), with about 2°C warming. The warming effect on soil microbial communities is linked to a wider range of factors than the temperature alone (Zhang B. et al., 2014). Warming likely influences the microbial communities directly by increasing the temperature and indirectly by changing the soil properties (Bardgett et al., 2008), and it occurred in this study. Firstly, Pearson’s correlation analysis showed significant relationships between the dominant microbial groups (phylum and genus level) and temperature (Supplementary Table S6). Then in the S1, variations in the abundance of some microbial groups were associated with the treatment-induced variations in carbon, nitrogen contents, and pH, while in the S2 and S3, the observed variations in abundance were associated only with changes in soil pH (Table 2 and Supplementary Table S6). The indirect change in soil properties may be the most important (Xiong et al., 2014), and in the present study, we found that the carbon and nitrogen contents and pH were the main driving factors for soil prokaryotic community structure.

Exposure time for warming is very important, which can affect the response of soil microbial communities to simulated climate change (Rinnan et al., 2007; Lamb et al., 2011). One study demonstrated that more than 10 years was needed to study the response of soil microbial communities to warming, supported by data showing that a 15-year warming period led to significant changes, while the 5-, 6-, and 10-year treatments did not (Rinnan et al., 2007). Lamb et al. (2011) found that a high arctic soil ecosystem resisted 16 years of warming; the aboveground community responded to the warming, but the soil microbial community, chemistry, and biochemistry did not. In our short-term study, we observed abundance variations in only some of the dominant groups. With longer-term monitoring, we may find more significant variations in the soil microbial community structure and composition, as well as gradual aboveground changes. For high arctic ecosystems, it could take many decades for this resistance to subside under persistent climate change. In some special ecosystems, such as the high arctic or plateaus, long-term monitoring will be very important to explore the response of soil microbial communities to warming.

### The Response of Soil Prokaryotic Community to Simulated Climate Change Varied With Three Water Table Peatlands

Soil prokaryotes in the S2 exhibited the largest change in prokaryotic community abundances and diversity index, indicating that they were more sensitive to the simulated climate change (Tables 1, 2). This may be due to the frequent dry-rewetting cycles of the peatlands. Studies have shown that dry-rewetting cycles may result in pulses of soil carbon and nitrogen contents from microbial intracellular solutes (Halverson et al., 2000), soil aggregate disruption, and cracking release (Miller et al., 2005). Soil microbial activity can be enhanced by organic amendments (Ros et al., 2003). The S2 was the only area with the dry-rewetting cycle, and sufficient substrates from this cycle may enhance the soil microbial activity. Additionally, the dry-rewetting cycle caused by water table fluctuations and the accompanying changes in oxygen and nutrient availability may provide a wide range of ecological niches for diverse microbe and their coexistence. This could explain the comparatively greater prokaryotic diversity in the S2 (Supplementary Table S3). The S3 may be more capable of defending itself against the effects of climate change than the S1, as it had higher soil microbial diversity and stable soil properties in the climate change treatments. This was supported by the fact that, in the S3 climate change treatments, the dominant groups (phylum and genus level) changed the least, and there were no significant variations in the prokaryotic diversity or richness index (Tables 1, 2).

### The Main Determinants of the Overall Soil Prokaryotic Community Structure

The RDA and PerMANOVA test showed a distinct pattern of the prokaryotic communities by the three water table peatlands rather than by the simulated climate change treatments (Figure 3 and Supplementary Table S4). This result was consistent with previous studies showing that site factors exert a strong effect on soil community structure (Walker et al., 2008; Corneo et al., 2013).

In our study, several factors may have contributed to the variations in overall prokaryotic community structure across three water table peatlands. RDA revealed that the environmental variables contributed to the most variation in prokaryotic community structure and that the peatland water tables and soil properties were the main determinants of prokaryotic communities. This was comparable to the results of other studies, where the soil water content and organic carbon availability were found to be the major determinants of soil microbial community structure (Drenovsky et al., 2004). We found significant differences in soil properties among three water table peatlands, and the simulated climate change treatments just significantly affected some indexes of the soil properties (Supplementary Table S1). These results indicated that soil property variations caused by simulated climate change treatments were so small that any effect on the overall microbial community structure was likely masked by the larger background variation in soil properties among the peatlands. This was consistent with a prior study, which documented a high magnitude of background variation in the microbial community that appeared to constrain the response to treatments (Gutknecht et al., 2012).

### Functional Microorganisms in the Carbon and Nitrogen Cycles

Methanogenic archaea play an important role in methanogenesis (Joblin, 2005), and all the Methanogenic archaea are in the phylum Euryarchaeota (Amils, 2011). Methane-oxidizing bacteria consume methane (a potent greenhouse gas) and are presently limited to Proteobacteria (Euzéby, 1997), Verrucomicrobia (Van Teeseling et al., 2014), and NC10 (Ettwig et al., 2010). In all three water table peatland areas, the abundances of Euryarchaeota, Proteobacteria, Verrucomicrobia, and *Methanobacterium* were not affected by the treatments, indicating that the simulated climate change conditions did not affect methane production and aerobic oxidation. The relative abundances of NC10 and Nitrospirae varied among the three water table peatlands, indicating that water table change may affect ammonia oxidation and the anaerobic oxidation of methane.

## Conclusion

Small-amplitude (0.23–2.4°C) and short-term (1 year) warming changed the microbial diversity and abundances of some of the groups (phylum and genus level) in the different water table peatlands, and soil prokaryotes in peatland with dry-rewetting cycle event were most sensitive to the simulated climate change. The peatland site factors (water table and soil properties) exert a stronger effect on the overall soil prokaryotic community structure compared to stimulated climate change, and the ammonia oxidation and anaerobic oxidation of methane may vary among three water table peatlands.

## Data Availability Statement

The raw data are deposited in the NCBI Sequence Read Archive with the accession number PRJNA314179.

## Author Contributions

NW, HC, and WL performed the design of the initial studies. WL designed the experiments and wrote the draft manuscript. HC revised the manuscript. ZY and GY performed the sampling work. JR and YH help to performed the laboratory work. All the authors have read and approved the final draft of the article.

## Conflict of Interest

The authors declare that the research was conducted in the absence of any commercial or financial relationships that could be construed as a potential conflict of interest.

## Footnotes

1 https://pyro.cme.msu.edu/classifier/form.spr

2 http://folk.uio.no/ohammer/past/
