# Supplementary material for: Variation in the Soil Prokaryotic Community Under Simulated Warming and Rainfall Reduction in Different Water Table Peatlands of the Zoige Plateau
Source: Front Microbiol. 2020 Mar 18;11:343. doi: 10.3389/fmicb.2020.00343 (PMC7093333; doi:10.3389/fmicb.2020.00343)
Supplement: Supplementary file 1 [file Data_Sheet_1.pdf]

Fig.S1. Water tables of three selected peatlands in 2012-2014.

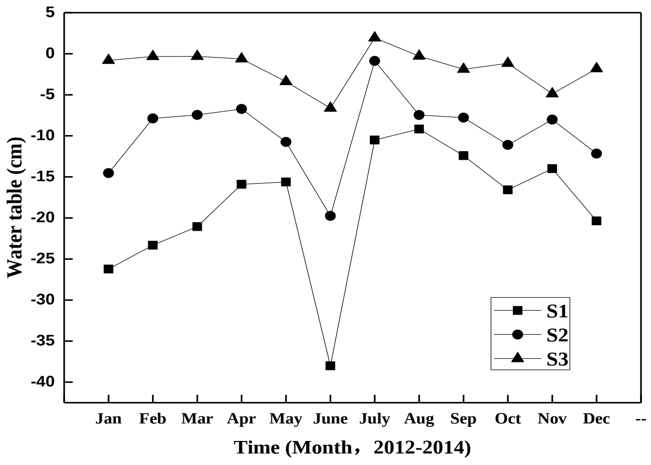

Table S1 Variation of environmental factors under simulated climate change treatments in peatlands of different water levels

Peatlands	S1				S2				S3			
Treatments	CK	W	R	WR	CK	W	R	WR	CK	W	R	WR
Soil temperature (°C)	-0.20	0.76	0.03	2.20	0.07	1.68	0.39	1.85	-0.61	0.81	-0.01	0.71
Moisture (%)	42	33	29	38	55	50	61	54	63	61	60	62
pH	5.67±0.06aA	5.95±0.03bA	5.82±0.09cA	5.78±0.01cA	5.93±0.04aA	6.06±0.07bB	6.20±0.03cB	5.82±0.09dA	5.52±0.03aB	5.68±0.02bC	5.71±0.01bcdC	5.74±0.05dA
Total carbon (%)	19.32±0.07aA	31.38±0.31bA	25.85±0.15cA	26.52±0.24cA	33.96±0.12aB	26.78±0.12aA	26.63±0.08aA	24.47±0.30aA	9.38±0.03aC	9.79±0.06aB	9.67±0.04aB	10.15±0.25aB
Total nitrogen (%)	1.45±0.00aA	2.19±0.02bA	1.70±0.02cA	1.79±0.00cA	2.30±0.02aB	1.90±0.03aA	1.88±0.01aA	1.82±0.02aA	0.67±0.00aC	0.71±0.00aB	0.70±0.00aB	0.73±0.02aB

CK: no warming or rainfall reduction; W: warming; R: 20% rainfall reduction; WR: warming + 20% rainfall reduction. Lowercase letters stand for the differences caused by simulated climate change treatments within peatlands of the same degradation level. Uppercase letters stand for the differences of the result of the same climate change treatment in the three levels of peatland degradation. All data are presented as means ± SD.

Table S2 Relative abundance (%) of the top 13 known genera and their respective phylogenetic affiliations

Taxon	S1CK	S1W	S1R	S1WR	S2CK	S2W	S2R	S2WR	S3CK	S3W	S3R	S3WR	T1	T2
Methanobacterium	0.14±0.04	0.40±0.12	0.29±0.06	0.45±0.14	3.13±1.17	0.75±0.51	3.79±1.60	1.24±0.61	0.84±0.12	1.48±0.45	1.50±0.72	1.34±0.60	Root	Archaea
Candidatus Solibacter	1.30±0.15	1.01±0.36	0.75±0.33	1.00±0.05	1.85±0.28	0.77±0.19	1.01±0.08	0.94±0.03	0.83±0.20	1.01±0.06	0.54±0.12	1.14±0.26	Root	Bacteria
Arthrobacter	0.03±0.01	0.15±0.11	0.02±0.01	0.07±0.02	0.21±0.03	0.42±0.38	0.45±0.13	0.05±0.01	1.27±0.08	0.92±0.58	0.64±0.36	0.48±0.16	Root	Bacteria
Hyphomicrobium	1.10±0.28	0.73±0.16	0.86±0.26	1.40±0.10	0.39±0.07	0.30±0.05	0.42±0.06	0.72±0.06	0.29±0.05	0.36±0.04	0.36±0.07	0.50±0.06	Root	Bacteria
Rhodoplanes	0.83±0.16	0.71±0.17	1.01±0.39	1.46±0.31	0.59±0.03	0.62±0.05	0.59±0.12	0.53±0.08	0.22±0.08	0.44±0.11	0.35±0.08	0.77±0.15	Root	Bacteria
Anaeromyxobacter	0.32±0.03	0.21±0.06	0.19±0.04	0.22±0.01	0.56±0.03	0.18±0.06	0.89±0.46	0.13±0.03	0.46±0.11	0.54±0.08	0.52±0.10	0.54±0.07	Root	Bacteria
Desulfobacca	0.20±0.05	0.06±0.02	0.09±0.03	0.16±0.08	0.00±0.00	0.02±0.00	0.02±0.00	0.05±0.04	0.13±0.01	0.19±0.06	0.25±0.03	0.24±0.05	Root	Bacteria
Desulfomonile	0.21±0.03	0.13±0.07	0.19±0.10	0.23±0.06	0.08±0.03	0.12±0.04	0.10±0.04	0.28±0.08	0.04±0.02	0.10±0.02	0.06±0.02	0.05±0.02	Root	Bacteria
Cellvibrio	0.12±0.01	0.19±0.08	0.05±0.04	0.05±0.02	0.01±0.01	0.01±0.01	0.01±0.01	0.00±0.00	0.01±0.00	0.05±0.04	0.00±0.00	0.03±0.01	Root	Bacteria
Pseudomonas	0.27±0.11	0.45±0.10	0.20±0.08	0.26±0.10	0.05±0.03	0.06±0.01	0.19±0.14	0.22±0.10	0.18±0.03	0.10±0.04	0.13±0.02	0.19±0.06	Root	Bacteria
Steroidobacter	0.10±0.02	0.20±0.04	0.01±0.01	0.03±0.02	0.11±0.01	0.01±0.00	0.02±0.01	0.01±0.01	0.04±0.01	0.02±0.00	0.05±0.02	0.04±0.02	Root	Bacteria
Luteolibacter	0.11±0.01	0.15±0.08	0.10±0.04	0.06±0.02	0.11±0.01	0.13±0.03	0.09±0.04	0.04±0.01	0.15±0.06	0.06±0.03	0.06±0.03	0.07±0.02	Root	Bacteria
DA101	5.66±2.09	1.59±0.32	3.18±1.26	2.52±0.83	0.37±0.07	1.07±0.47	0.43±0.08	0.51±0.05	0.19±0.06	0.25±0.06	0.15±0.08	0.35±0.13	Root	Bacteria

CK: no warming or 20% rainfall reduction; W: warming; R: 20% rainfall reduction; WR = warming + 20% rainfall reduction. S1: All data are presented as means ± SD.

Table S3 Prokaryotic diversity indices based on 97% identity of 16S rRNA gene sequences and 10,280 reads per sample

Peatlands	S1				S2				S3			
Treatments	CK	W	R	WR	CK	W	R	WR	CK	W	R	WR
Chao1 estimator of richness	6161.37±585.39	8236.12±581.71	5291.02±602.89	5478.31±662.24	7497.16±424.75	6558.18±178.63	7602.57±218.80	6559.06±493.04	7542.02±285.62	7432.34±198.05	7603.84±141.66	7876.90±226.71
observed-species	2974.57±169.12	3908.50±212.21	2729.20±207.73	2758.67±251.44	3819.57±192.74	3105.23±39.77	3581.37±115.52	3055.93±189.43	3517.43±97.87	3431.67±63.72	3541.90±161.92	3614.20±122.18
Shannon’s diversity index	10.18±0.00	10.93±0.14	9.84±0.25	9.77±0.40	10.93±0.15	10.19±0.05	10.69±0.12	11.21±0.18	10.61±0.12	10.55±0.06	10.49±0.18	10.65±0.11

CK: no warming or 20% rainfall reduction; W: warming; R: 20% rainfall reduction; WR = warming + 20% rainfall reduction. All data are presented as means ± SD.

Table S4 PerMANOVA test of the differences in prokaryotic community structure based on Unifrac distance measures (permutation: 9999)

	S1	S2	S3
S1	0.0000	0.0001	0.0001
S2	0.0001	0.0000	0.0001
S3	0.0001	0.0001	0.0000

Table S5 Pearson's correlation among environmental variables

	Total Carbon	Total Nitrogen	pH	Water Table
Total Nitrogen	0.995**			
pH	0.705**	0.713**		
Water Table	-0.646*	-0.636**	-0.252	
Soil 5 cm Temperature	0.246	0.244	0.384*	-0.196

** $p<0.01$; * $p<0.05$.

Table S6 Pearson's correlation of dominant phyla and genera with environmental variables

	Total Carbon	Total Nitrogen	pH	Water Table	Soil Temperature
(a) phylum					
Crenarchaeota	0.000	0.043	0.280	0.226	0.147
Euryarchaeota	-0.115	-0.092	0.221	0.542**	0.040
Parvarchaeota	-0.426**	-0.421*	-0.103	0.536**	0.197
Acidobacteria	0.416*	0.448*	0.208	-0.727**	-0.028
Actinobacteria	-0.003	-0.018	-0.022	0.083	-0.108
Armatimonadetes	-0.133	-0.149	-0.149	0.325	-0.356*
Bacteroidetes	-0.396*	-0.398*	-0.347*	0.573**	-0.405*
Chlorobi	0.165	0.211	0.323	0.186	0.536**
Chloroflexi	0.116	0.101	0.197	0.032	0.052
Cyanobacteria	-0.039	-0.068	-0.092	0.312	-0.277
Elusimicrobia	-0.163	-0.129	0.029	0.333*	0.037
Fibrobacteres	0.130	0.136	0.066	-0.082	-0.025
Firmicutes	-0.122	-0.159	-0.158	-0.110	0.341*
Gemmatimonadetes	0.192	0.150	0.042	-0.362*	0.058
NC10	0.298	0.332*	0.127	-0.624**	0.098
Nitrospirae	0.208	0.215	-0.038	-0.731**	-0.001
OD1	-0.488**	-0.485**	-0.290	0.532**	-0.035
OP8	0.234	0.263	0.371*	0.033	0.128
Planctomycetes	0.254	0.243	0.218	-0.127	0.249
Proteobacteria	-0.175	-0.175	-0.318	0.030	-0.171
Spirochaetes	0.179	0.197	0.128	-0.106	0.538**
Verrucomicrobia	0.091	0.103	-0.085	-0.479**	-0.156
WS3	0.374*	0.386*	0.363*	-0.353*	0.074
TM7	-0.003	-0.020	-0.151	0.245	-0.389*
(b) genera					
Methanobacterium	0.091	0.093	0.345*	0.319	0.148
Candidatus Solibacter	0.302	0.305	0.085	-0.099	-0.151
Arthrobacter	-0.485**	-0.487**	-0.315	0.595**	-0.343*
Hyphomicrobium	0.228	0.223	-0.107	-0.710**	0.368*
Rhodoplanes	0.308	0.285	0.131	-0.583**	0.392*
Anaeromyxobacter	-0.182	-0.186	0.115	0.389*	-0.039
Desulfobacca	-0.705**	-0.708**	-0.620**	0.220	-0.114
Desulfomonile	0.186	0.207	-0.001	-0.488**	0.369*
Cellvibrio	0.216	0.220	0.015	-0.528**	-0.239
Pseudomonas	0.129	0.131	-0.037	-0.428**	0.071
Steroidobacter	0.323	0.322	0.026	-0.351*	-0.410*
Luteolibacter	0.167	0.153	0.089	-0.139	-0.384*
DA101	0.176	0.184	-0.077	-0.659**	-0.060

** $p<0.01$, * $p<0.05$.
